# Sustainable Thermoelectric Composites: A Study of Bi_2_Te_3_-Filled Biobased Resin

**DOI:** 10.3390/ma18153453

**Published:** 2025-07-23

**Authors:** Luca Ferretti, Pietro Russo, Jessica Passaro, Francesca Nanni, Saverio D’Ascoli, Francesco Fabbrocino, Mario Bragaglia

**Affiliations:** 1 Department of Enterprise Engineering “Mario Lucertini”, University of Rome “Tor Vergata” and INSTM RU Roma-Tor Vergata, 00133 Rome, Italy; luca.ferretti.29@students.uniroma2.eu (L.F.); fnanni@ing.uniroma2.it (F.N.); 2 Institute for Polymers, Composites and Biomaterials, National Council of Research, 80078 Pozzuoli, Italy; pietro.russo@ipcb.cnr.it (P.R.); jessica.passaro@cnr.it (J.P.); 3 Department of Engineering, Pegaso Telematic University, 80143 Naples, Italy; saverio.dascoli@unipegaso.it (S.D.); francesco.fabbrocino@unipegaso.it (F.F.)

**Keywords:** thermoelectric, bio-composites, 3D printing, energy harvesting

## Abstract

In this work, bio-based thermoelectric composites were developed using acrylated epoxidized soybean oil (AESO) as the polymer matrix and bismuth telluride (Bi_2_Te_3_) as the thermoelectric filler. The materials were formulated for both UV-curing and thermal-curing processes, with a focus on Digital Light Processing (DLP) 3D printing. Although UV curing proved ineffective at high filler concentrations due to the light opacity of Bi_2_Te_3_, thermal curing enabled the fabrication of stable, homogeneously dispersed composites. The samples were thoroughly characterized through rheology, FTIR, TGA, XRD, SEM, and density measurements. Thermoelectric performance was assessed under a 70 °C temperature gradient, with Seebeck coefficients reaching up to 51 µV/K. Accelerated chemical degradation studies in basic media confirmed the degradability of the matrix. The results demonstrate the feasibility of combining additive manufacturing with sustainable materials for low-power thermoelectric energy harvesting applications.

## 1. Introduction

As the climate crisis continues to intensify due to the anthropogenic greenhouse effect and the extensive use of fossil fuels, the development of renewable and environmentally friendly energy sources represents one of the most urgent challenges facing humanity [1]. At the same time, the disposal of petroleum-derived materials such as plastics poses additional environmental concerns. Consequently, the search for bio-based and biocompatible materials has become essential to mitigating the ecological footprint of fossil fuel usage [2]. A promising approach to address these issues involves the use of thermoelectric phenomena such as the Seebeck and Peltier effects [3]. These effects enable direct conversion between thermal and electrical energy, providing a solid-state solution with no moving parts or chemical reactions. In particular, the Seebeck effect refers to the generation of a voltage difference when two dissimilar conductors or semiconductors are joined at two junctions maintained at different temperatures. The resulting voltage (ΔV) is proportional to the temperature gradient (ΔT) and the material’s intrinsic Seebeck coefficient (S), defined as [4]

(1) $$S\left[\frac{\mu V}{K}\right]=\frac{\Delta V}{\Delta T}$$

Metals typically exhibit low Seebeck coefficients (1–10 µV/K), whereas semiconductors can reach values around 100 µV/K [5]. Although S generally exhibits a nonlinear dependence on temperature, it can be approximated as linear over small temperature ranges [6,7]. The field of thermoelectricity enables new strategies for energy harvesting, particularly by recovering waste heat from industrial processes or thermal engines. These technologies rely solely on the intrinsic properties of materials, eliminating the need for mechanical or chemical systems. The use of thermoelectric devices to generate power from a temperature difference represents a sustainable and environmentally friendly energy solution. These devices are reliable, long-lasting owing to the absence of moving parts, and operate silently [6]. Despite these advantages, their current applications remain limited to specialized fields such as the medical, military, and aerospace sectors, or to remote environments, due to their relatively high cost and limited efficiency. Electricity generation occurs when a thermoelectric module is placed between a heat source and a heat sink, allowing thermal energy to flow through the device. If a sufficient temperature gradient is maintained across the module, a voltage is generated [8]. Low-power thermoelectric generators (TEGs) can exploit even small temperature differences. For instance, Seiko and Citizen have incorporated TEGs into wristwatches powered by the heat from the human body compared with ambient air, achieving 300 mV from a ∆T of just 1.5 K [9]. To further enhance the performance of thermoelectric materials and expand their applicability into emerging fields requiring specific functional properties, thin-film technologies have been extensively explored in recent years. These systems enable the development of flexible, lightweight, and miniaturized devices, offering mechanical adaptability while maintaining thermoelectric functionality. In particular, thin films based on Ag_2_Q (Q = S, Se, Te) compounds have demonstrated Seebeck coefficients and overall thermoelectric performance comparable to bulk materials. These compounds have attracted growing interest due to their doping versatility, which allows fine-tuning of carrier concentration, transport properties, and texturing. As a result, thin-film thermoelectrics represent a promising route toward wearable and flexible energy-harvesting devices with competitive efficiency and integration potential in next-generation electronics [10,11,12]. Also, as alternatives to conventional batteries, low-power TEGs offer several advantages. Batteries, although increasingly efficient, still pose environmental and safety concerns due to their limited lifespan and toxic materials, including nickel, zinc, sulfuric acid (which is also highly corrosive), and mercury [6]. TEGs based on Bi_2_Te_3_, in particular, provide a compact, cost-effective, and efficient solution. When fabricated as thin films, they allow the p–n couples of the module to be integrated into limited spaces [8]. Although thermoelectric devices struggle to compete with high-output energy systems due to their low efficiency, they remain highly suitable for low-power generation. There is growing interest in their application for harvesting waste heat from industrial processes, solar radiation, and vehicle exhausts. Automotive manufacturers such as Volvo, Ford, and BMW are actively investing in TEGs capable of converting exhaust heat, typically around 200 °C, into usable electricity, contributing to fuel efficiency improvements of over 16% [8]. Bismuth telluride (Bi_2_Te_3_) and its alloys are, to date, the most well-performing and widely used materials for thermoelectric applications at room temperature. They are extensively employed both as power generators, especially for sources operating at moderate temperatures [13], and as Peltier coolers due to their high stability and efficiency [14]. Bi_2_Te_3_ exhibits the highest figure of merit (ZT), a dimensionless parameter used to compare the performance of thermoelectric materials [15], operating at ambient temperatures, owing to its unique combination of low effective mass, high charge carrier mobility, strong band degeneracy, and low lattice thermal conductivity [14].

The latter property is largely attributed to the presence of tellurium, the heaviest non-radioactive chalcogen, which contributes to lowering lattice thermal conductivity: a key requirement for achieving high ZT values [16]. Moreover, tellurium-containing compounds are generally less ionic, enhancing carrier mobility and thereby improving electrical conductivity and overall thermoelectric efficiency [16]. Structurally, Bi_2_Te_3_ crystallizes in a rhombohedral–hexagonal symmetry, with alternating layers of Bi and Te atoms bound by strong covalent bonds, while adjacent Te layers are connected by weak van der Waals forces. This peculiar structure results in significant anisotropy of thermal and electrical properties: thermal conductivity is twice as high along the basal planes compared with the perpendicular direction, and electrical conductivity is six times greater along the basal planes in n-type Bi_2_Te_3_ (and three times greater in p-type material) [14]. Conversely, the Seebeck coefficient remains approximately isotropic, as it is primarily determined by the uniform layer density. Compared with other chalcogenides, Bi_2_Te_3_ also displays a band gap that decreases with increasing temperature, a typical behavior of covalent semiconductors [17]. Owing to these favorable properties, Bi_2_Te_3_-based thermoelectric modules have been employed in a variety of applications, particularly in scenarios where compactness, durability, and energy autonomy are critical. Thermoelectric generators (TEGs) based on Bi_2_Te_3_ enable power generation from small temperature differences, making them suitable for wearable electronics, remote sensors, and medical implants, where they can replace traditional batteries with a safer, long-lasting, and environmentally friendly alternative [6,8]. The ongoing study of high-performance Bi_2_Te_3_-based materials has revealed several strategies to improve thermoelectric properties. Among them, doping with donor or acceptor impurities helps optimize the carrier concentration to maximize the Seebeck coefficient without severely impacting electrical conductivity. The use of solid solutions based on selenium (Se) and antimony (Sb) has been effective in reducing lattice thermal conductivity via enhanced phonon scattering while preserving high carrier mobility [14]. Additional approaches include nanostructuring, which selectively decreases lattice thermal conductivity by promoting phonon scattering at grain boundaries, and engineering the band gap to modify the inertial effective mass, balancing between carrier mobility and Seebeck coefficient optimization [18]. Typically, TEGs are produced via the melting and solidification technique or the powder metallurgy route [19]; nevertheless, these processes are energy-intensive, and the carbon footprint is high. A possible solution for the production of thermoelectric composites relies on the use of a polymer matrix filled with a powder of TEGs [20]. On the other hand, due to the increasing environmental concerns associated with traditional plastics, significant attention has to be directed toward the development of bio-based polymers partially or entirely derived from renewable resources [21,22]. In this specific context, the use of a bio-based, potentially biodegradable resin could provide two key advantages that go beyond general sustainability. First, it would allow for the production of thermoelectric composites with a reduced carbon footprint and overall environmental impact [23]. Second, it would enable the potential recovery and reuse of the thermoelectric filler (Bi_2_Te_3_) at the end of the composite’s service life through degradation or removal of the polymer matrix. This approach aligns with circular economy principles, minimizing material waste and improving resource efficiency. Such recyclability would be particularly valuable in constrained environments like space missions, where material reuse and self-sufficiency are critical [24]. Vegetable oils have emerged as attractive raw materials owing to their biodegradability, wide availability, and reactive chemical structures. Soybean oil, in particular, is one of the most widely produced and utilized vegetable oils globally. Composed predominantly of triglycerides rich in unsaturated fatty acids, soybean oil provides multiple reactive C=C double bonds. Through epoxidation [25], these unsaturations are converted into reactive epoxide groups, which can then be acrylated, producing acrylated epoxidized soybean oil (AESO) [26]. AESO serves as a key building block for UV-curable resins and other advanced polymeric materials. The use of acrylated soybean oil thus represents a strategic approach toward sustainable materials, aligning with the principles of green chemistry while maintaining the functional and mechanical characteristics necessary for industrial applications and allowing also for additive manufacturing (AM) processes [27], i.e., vat photopolymerization methods such as Stereolithography (SLA) and Digital Light Processing (DLP), that selectively cure liquid photopolymers using UV light [28,29]. Epoxidized linseed oil and acrylated epoxidized soybean oil (AESO) have been successfully used to produce UV-curable resins without additional photoinitiators [30,31].

This is made possible by the intrinsic reactivity of the acrylate and epoxide functional groups, which can absorb UV light and initiate polymerization independently or in the presence of natural co-initiators. Such systems reduce the need for synthetic photoinitiators, lowering toxicity, cost, and formulation complexity while improving the overall environmental footprint of the resin. In this work, we report on the development of a sustainable thermoelectric bio-composite consisting of a bio-based matrix filled with bismuth telluride (Bi_2_Te_3_), a well-known thermoelectric material. The matrix is derived from soybean oil, which is abundant, low-cost, and renewable. The composite was formulated to allow easy processing and to ensure effective thermoelectric performance. In particular, we explored the feasibility of using 3D printing techniques, including both photopolymerization and thermal curing, for the fabrication of the bio-resin. Comprehensive characterization of the composite materials was conducted, focusing on their thermoelectric behavior, microstructure, and stability. The analyses included scanning electron microscopy (SEM), X-ray diffraction (XRD), Fourier transform infrared spectroscopy (FTIR), thermogravimetric analysis (TGA), as well as density and degradation studies.

## 2. Experiments

### 2.1. Raw Materials and Preliminary Characterization

The resin systems developed in this work are based on epoxidized soybean oil (AESO), supplied by Sigma-Aldrich (St. Louis, MO, USA), which serves as the bio-based component of the formulation. AESO is a renewable material with a density of 1.04 g/mL at 25 °C, a flash point of 113 °C, and a refractive index of 1.484. To tailor the mechanical and surface properties of the final polymer, poly (ethylene glycol) diacrylate (PEGDA) and pentaerythritol triacrylate (PETA) were incorporated. PEGDA is a hydrophilic monomer and crosslinker with a viscosity ranging from 40 to 75 cP at 25 °C, which enhances flexibility, adhesion, and chemical and impact resistance. PETA, on the other hand, contributes to improved surface morphology and forms a dense crosslinking network, increasing abrasion resistance and the mechanical strength of the polymer. Each formulation includes a constant amount of thermoelectric filler composed of bismuth telluride (Bi_2_Te_3_), supplied by Alfa Aesar (Thermo Fisher Scientific, Waltham, MA, USA). This material, with a purity of 99.98% and a melting point of 573 °C, is widely recognized for its high thermoelectric performance. Two different initiators were used, depending on the chosen polymerization method:

- Bis (2,4,6-trimethylbenzoyl)-phenylphosphine oxide (BAPO) was employed in the case of photopolymerization. Supplied by Sigma-Aldrich, BAPO has a melting point of 131–135 °C, a purity of 97%, and is active in the UV range of 350–420 nm;
- Dicumyl peroxide (DCP) was used for thermal curing. Also obtained from Sigma-Aldrich, DCP features high thermal stability due to the steric hindrance from adjacent substituents near the peroxide group. Upon heating, it decomposes via homolytic cleavage in the peroxide bond region, initiating the crosslinking reaction.

### 2.2. Preparation and Preliminary Characterization of Resin Batches

The different resin batches were prepared by mixing AESO, PETA, and PEGDA in a beaker equipped with a magnetic stirrer operating at approximately 150 rpm. The Bi_2_Te_3_ filler was first dispersed in acetone using a 1:1 mass-to-volume ratio (1 g of filler per 1 mL of acetone). The dispersion was sonicated for 30 min using an ultrasonic bath (ElmaSonic S 30H, Singen, Germany, operating at 37 kHz) to promote uniform distribution and prevent agglomeration. After sonication, the filler suspension was added to the resin mixture. The acetone was then allowed to evaporate under continuous stirring to ensure homogeneity of the composite. Following solvent evaporation, the batches were divided into two groups according to the type of polymerization initiator added: BAPO for photopolymerization and DCP for thermal curing. During this phase, particular attention was given to protecting the BAPO-containing batches from light exposure to prevent premature crosslinking. The several thermoelectric formulations differed based on the AESO–PETA–PEGDA ratios, the filler weight percentage, and the initiator concentration, as summarized in Table 1.

To evaluate the printability of the BAPO-containing resin formulations, rheological tests were performed on all batches. Measurements were conducted using a Malvern Kinexus Lab+ rheometer (Netzsch, Bayern, Germany) in a plate–cone (40 mm, α = 1°, gap 0.1 mm) configuration. The viscosity was evaluated by varying the shear rate between 0.01 and 100 s^−1^ at room temperature. Subsequently, to verify the presence and structural integrity of the bismuth telluride (Bi_2_Te_3_) filler after incorporation into the resins, X-ray diffraction (XRD) analysis was performed. Both the Bi_2_Te_3_ powder and the composite resins were examined using a Philips X’Pert 1710 diffractometer (Philips, Amsterdam, The Netherlands) over a 2θ range of 10–90°. The measurements were carried out under the following conditions: Cu Kα radiation (λ = 1.5408 Å), an accelerating voltage of 40 kV, a current of 40 mA, a step size of 0.02°, and a dwell time of 2 s per step.

### 2.3. Crosslinking Tests and 3D Printing of BAPO-Based Resins

Preliminary crosslinking tests were conducted by depositing a single drop of each batch either on a glass slide or inside a Teflon mold (30 × 12 × 2 mm) to form a thin layer. For the BAPO-containing batches, a UV lamp (Aibecy, 60 W, 405 nm) was positioned over the samples to determine the approximate curing time required for subsequent 3D printing. For the DCP-containing batches, the molds were placed in an oven at 100 °C. In addition, the 6:3:1-75-20D resin was also tested at an elevated curing temperature of 120 °C. The BAPO-based formulations were further evaluated using a commercial DLP 3D printer, the Elegoo Mars 2 Pro (Elegoo Inc., Shenzhen, China), featuring a build volume of 143 × 90 × 175 mm^3^. The digital models were prepared and sliced using Chitubox software, with the printing parameters summarized in Table 2.

### 2.4. Physical, Chemical, and Morphological Characterization

Density measurements were performed following ASTM D792 [26,32], using a pycnometer (Sartorius, Göttingen, Germany) based on the Archimedes principle. All the produced samples were analyzed by Fourier transform infrared spectroscopy (FTIR) using a Cary 630 FTIR spectrometer (Agilent, Santa Clara, CA, USA). Spectra were collected in the range of 600–4000 cm^−1^ with a resolution of 4 cm^−1^, averaging 32 scans per measurement. Uncured resins were also tested under the same conditions to compare chemical differences pre- and post-curing. Thermogravimetric analysis (TGA) was conducted on the 3D-printed specimens using a Pyris 1 TGA analyzer (PerkinElmer, Waltham, MA, USA) under a nitrogen atmosphere (40 mL/min). The temperature program ranged from 25 °C to 700 °C with a heating rate of 10 °C/min. The morphology of the sample surfaces was investigated via field-emission scanning electron microscopy (FEG-SEM) using a Leo Supra-35 microscope (Zeiss, Oberkochen, Germany) operating at an accelerating voltage of 5 kV. In order to prevent charging effects on the non-conductive resin samples, a thin gold coating (~10 nm) was deposited prior to imaging. Hydrolytic degradation tests were carried out by immersing the specimens in 13 mL of 1 M NaOH solution (Carlo Erba Reagents, Cornaredo, Italy). The samples were incubated at 37 °C in a Memmert incubator. Degradation progress was monitored every 24 h, with gentle shaking before each observation, until complete dissolution of the polymer matrix.

### 2.5. Thermoelectric Measurements

**Figure 1:**
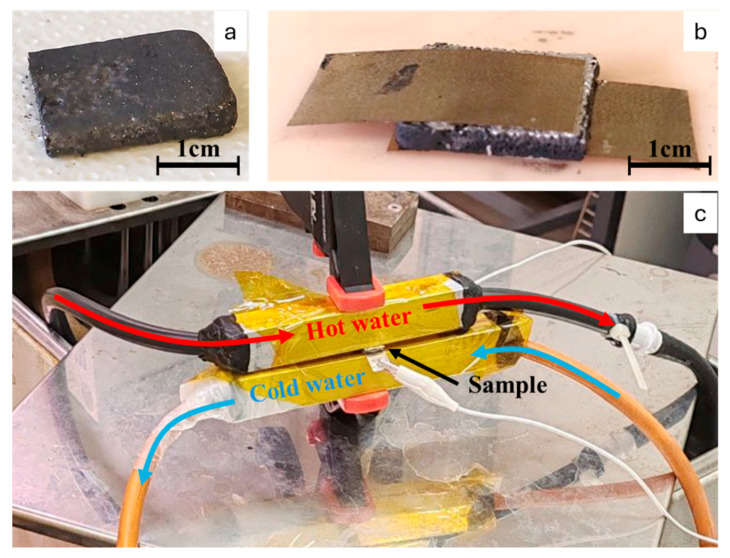
(**a**) The 6:3:1-75-20D specimen tested for thermoelectric properties. (**b**) Sample prepared between two Cu–Ag foil sheets attached with silver paste. (**c**) Assembled thermoelectric testing system. Red arrows represent the flow of the hot water, blue ones represent the flow of cold water, and the black one indicates the sample positioning.

To measure the thermoelectric properties of the samples, rectangular sections (15 × 12 × 2 mm) were cut from the cured specimens. The samples were washed in alcohol to remove any uncured resin residues, and then dried. As shown in Figure 1, on the two larger faces of each sample, a thin Cu–Ag foil (thickness: 150 µm) was applied. The adhesive used was a silver-based conductive epoxy resin (ICP9000, Henkel Adhesives, Düsseldorf, Germany). The assembled structure was then placed in an oven at 170 °C for 1 h to ensure complete crosslinking of the conductive epoxy. After curing, each specimen was positioned between two aluminum tubes (square cross-section, 2 cm side length, 2 mm wall thickness), which were, respectively, connected to hot- and cold-water circuits through rubber tubes.

The hot water was maintained at a constant temperature using a thermostatic bath (Julabo, Seelbach, Germany) with a temperature stability of ±0.15 °C and uniformity of ±0.01 °C. For the cold side, two experimental conditions were evaluated. In the first test, the cooling tubes were left exposed to ambient air. In the second test, cold water from an ice-water bath (maintained at 0 °C) was circulated through the tubes using a peristaltic pump. The specimen was secured between the two aluminum blocks using a vice, and electrical contacts were added to the Cu–Ag-coated surfaces for voltage monitoring. The electrical signal was recorded using a Keithley 2700 multimeter (Beaverton, OR, USA) (sensitivity: 100 nV). During testing, the temperature difference (ΔT) between the two faces and the corresponding potential difference (ΔV) were monitored simultaneously. To prevent short circuits, all electrical contacts were insulated with Kapton tape (thickness: 0.06 mm).

The Seebeck coefficient was calculated applying Equation (1), where ΔV is the averaged voltage over time and ΔT is the difference between the hot and the cold contacts. The figure of merit ZT was calculated following Equation (2), where ρ is the resistivity of the sample measured in Ω·m, Κ is the thermal conductivity measured in W/(K·m), S is the Seebeck coefficient, and T is the mean operating temperature of the sample equal to (T_hot_ + T_cold_)/2 [33].

(2) $$ZT\;=\frac{S^{2}}{\rho \times Κ}T$$

The sheet resistance Rs (measured in Ohms per square, Ω/□ or Ω/sq, equivalent to Ωm/m) was measured applying the van der Pauw method, contacting four probes on the perimeter of rectangular samples (15 × 10 × 3 mm) at four different points [34]. The resistivity was calculated using Equation (3), where t is the thickness of the sample.

(3) $$\rho =R_{S}\times t$$

The thermal conductivity Κ was evaluated through hot disk measurements following standard ISO 22007-2 2022 [35], (TPS 500 Hot Disk Instruments, Skövde, Sweden), applying 100 mW power for 10 s and using a thin sensor (3.2 mm diameter).

## 3. Results and Discussion

### 3.1. Bismuth Telluride Filler Phase Analysis and Morphology

**Figure 2:**
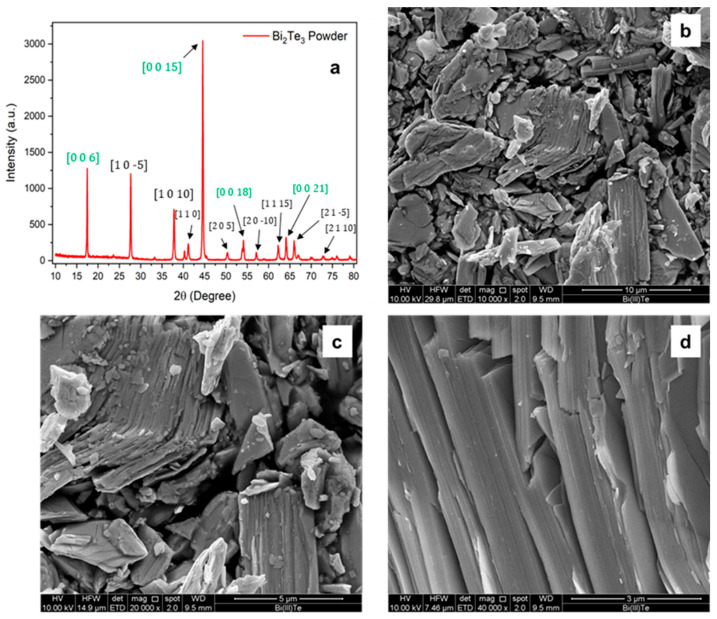
(**a**) XRD pattern of the Bi_2_Te_3_ powder and of the loaded resin. The green highlighted directions represent multiples of the main crystallographic direction [0 0 3]. (**b**–**d**) SEM images of the powder at higher magnification. Laminated pattern is visible following a certain direction.

The X-ray diffraction (XRD) patterns obtained for the bismuth telluride powder are shown in Figure 2a.

The diffraction peaks observed for the powder match those reported in the reference database JCPDS 96-901-1963, confirming the presence of Bi_2_Te_3_ with a hexagonal–rhombohedral crystal structure. The SEM analysis in Figure 2b–d reveals that, at low magnification, the Bi_2_Te_3_ powder appears as an agglomerate of particles with varying sizes, all exhibiting the characteristic lamellar morphology associated with van der Waals layered crystals. This morphology arises from the weak interlayer forces that allow easy cleavage along specific crystallographic planes. This layered structure becomes more evident at higher magnification, where step-like features and smooth facets are clearly observed. The highly aligned orientation of the steps within individual grains further supports the presence of a preferential crystallographic orientation, in agreement with the XRD results.

### 3.2. Rheological Behavior of Neat and Bi_2_Te_3_-Filled Resin

**Figure 3:**
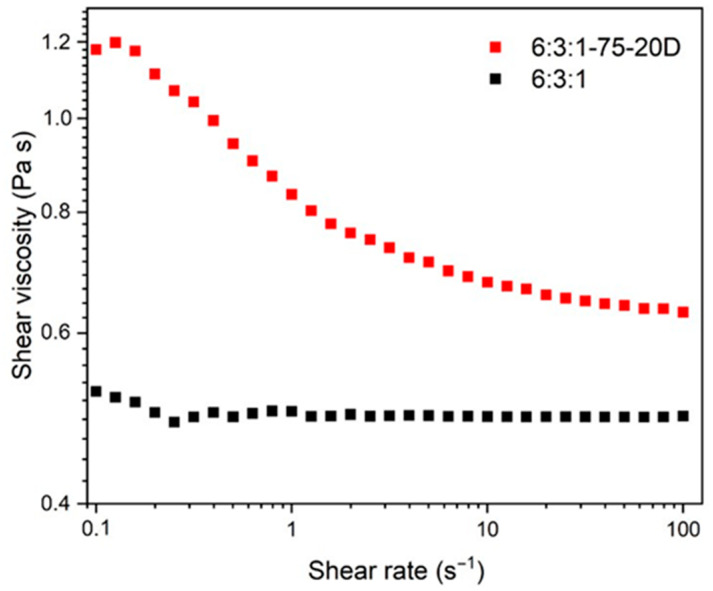
Rheological behavior of the resin formulations with (red) and without (black) Bi_2_Te_3_ filler.

The rheological analysis (Figure 3) shows that the loaded resins exhibit a shear-thinning behavior, with the viscosity decreasing from 1.2 Pa·s at a shear rate of 0.1 s^−1^ to 0.65 Pa·s at 100 s^−1^. According to the literature [36], the viscosity range required for a resin to be considered printable lies between 0.25 and 5 Pa·s. Therefore, the developed resins satisfy the printability requirements across the entire shear rate range investigated.

Furthermore, the neat AESO–PEGDA–PETA resin matrix behaves as a Newtonian fluid, exhibiting nearly constant viscosity across different shear rates [37]. This behavior is typical of low-molecular-weight, uncured resin systems where the polymer chains can move freely in solution without strong entanglement or secondary interactions. In contrast, the incorporation of Bi_2_Te_3_ powder into the resin drastically alters its flow behavior, passing from Newtonian to shear thinning, which is typical of polymer-filler systems [38,39]. The viscosity reduction with increasing shear rate can be ascribed to the disruption of the microstructure formed by the dispersed thermoelectric particles within the polymer matrix. At low shear rates, particle–particle interactions, hydrodynamic forces, and potential agglomerates increase the internal resistance to flow, resulting in high viscosity [40]. Increasing the shear rate, the applied stress is sufficient to break the particle network and align the particles along the flow direction, thereby reducing flow resistance. This disruption allows the polymer chains to move more freely, leading to a reduction in viscosity.

### 3.3. Photo Crosslinking and Thermal Crosslinking

**Figure 4:**
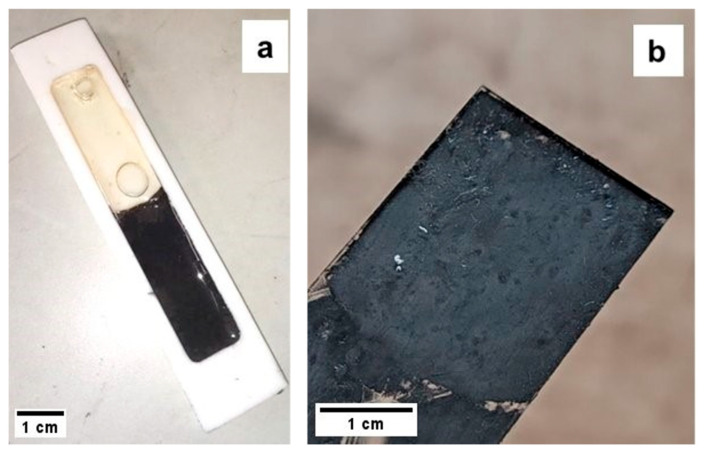
(**a**) The 6:3:1-75-20D sample inside a Teflon mold alongside the 6:3:1 resin, thermally cured at 100 °C; (**b**) the 5:5:1-50-1B sample cured under a UV lamp for 5 min.

The photocuring resins demonstrated behavior compatible with 3D-printing processes (Figure 4). Specifically, 5:5:1-25-1B and 5:5:1-50-1B fully cured in under 5 min, whereas 5:5:1-75-1B, 5:5:1-75-2B, and 6:3:1-75-1B required over 1 h to complete curing, indicating a significantly less efficient but still possible printing process. This slower curing is attributed to the UV-opacity of the filler (Bi_2_Te_3_), which leads to greater UV absorption at 405 nm and thus delayed crosslinking [41]. Given the expectation of poor thermoelectric performance for batches with filler contents below 50%, only the batches with a 6:3:1 AESO–PEGDA–PETA ratio were selected for 3D printing attempts. Among these, 6:3:1-75-2B showed the best curing performance, achieving polymerization in approximately 45 min. Despite using the parameters listed in Table 2, all batches ultimately failed the 3D printing tests, even after extending the exposure time per layer to 6 min. The failure mechanism was consistent across samples: while the printed layers adhered to each other, they failed to sufficiently stick to the build plate, indicating inadequate curing depth. This issue is attributed to the strong absorption of UV light by the Bi_2_Te_3_ filler, particularly in the near-UV range around 405 nm, which corresponds to the emission wavelength of the DLP printer used [42]. Bi_2_Te_3_ is known to exhibit a strong optical response even in the visible and near-UV spectrum. As reported in the literature, this material significantly attenuates UV irradiation due to its high absorption coefficient, which effectively prevents sufficient light penetration into the photopolymerizable resin matrix. Consequently, the photoinitiated crosslinking reaction remains confined to the resin surface and fails to establish robust adhesion with the build plate [43]. Although longer exposure times could potentially offset this limitation, the curing durations required would undermine the efficiency and throughput of the DLP process, making it impractical for additive manufacturing purposes. As a result, given the insufficient performance of the DLP process under these conditions, the focus was shifted toward thermal curing using DCP-based resins. Thermal curing tests on 6:3:1-75-20D were conducted at 100 °C and 120 °C, with curing times of approximately 150 min and 90 min, respectively. Comparison between the two samples showed that curing at 100 °C produced specimens with less porosity and reduced structural deformation, suggesting that 6:3:1-75-5D should also be cured at 100 °C to optimize quality. The 6:3:1-75-5D resin, when cured under these conditions, exhibited similar porosity levels to 6:3:1-75-20D, indicating that all these formulations can be considered suitable for subsequent testing.

### 3.4. Physical, Chemical, and Morphological Properties of Bi_2_Te_3_ Composites

The neat components used in the formulations PEGDA, PETA, AESO, and Bi_2_Te_3_ exhibit densities of 1.12 g/cm^3^, 1.19 g/cm^3^, 1.04 g/cm^3^, and 7.7 g/cm^3^, respectively. Applying the rule of mixtures, the theoretical density of the polymeric matrix is calculated to be approximately 3.10 g/cm^3^, while the theoretical density of the composite (including the filler) is approximately 3.08 g/cm^3^. The results of the experimental density measurements are reported in Table 3. As observed, the specimen cured at 120 °C exhibits a significantly lower density than expected, attributed to a high internal porosity, estimated at around 40%.

**Figure 5:**
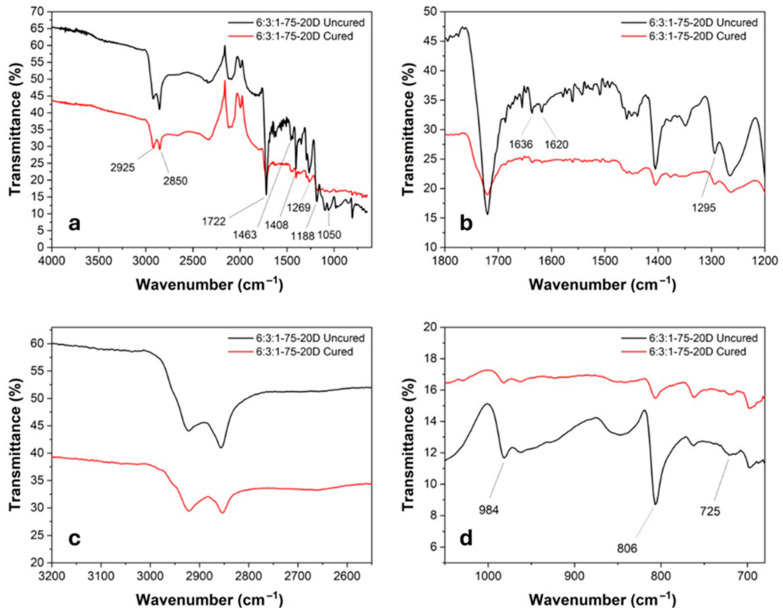
(**a**) General FTIR spectra of cured (red) and uncured (black) 6:3:1-75-20D with highlighted peaks; (**b**–**d**) detail of relevant peaks.

This porosity may result from micro-voids generated during the curing process, likely due to gas evolution associated with peroxide decomposition and insufficient matrix consolidation at higher temperatures. In contrast, the sample 6:3:1-75-20D cured at 100 °C reached a measured density of 3.60 g/cm^3^—well above the theoretical value. Although unexpected, this outcome may be attributed to a more efficient packing and reduced void content, resulting from the combination of lower curing temperature and higher DCP concentration. A denser crosslinked network tends to contract the available free volume, leading to shrinkage and densification of the matrix [37]. It is important to consider that the theoretical density is derived from the properties of the liquid-state components. Upon curing, the transition to a solid state involves molecular rearrangement, shrinkage, and changes in the packing density, which may cause deviations from the predicted values. A measured density higher than the theoretical value therefore suggests efficient polymer network formation and effective filler integration. It should also be noted that all the reference densities used in the calculation refer to the liquid phase of the respective materials; hence, post-curing structural changes and phase transitions may lead to deviations in the actual solid-state densities. These findings highlight how the curing parameters and initiator concentration can influence the internal structure and performance-related physical properties of thermoelectric composites. For the FTIR studies, the spectra of the liquid resin and the cured specimen from 6:3:1-75-20D were compared. As shown in Figure 5, the cured resin exhibits a general increase in peak intensity, attributed to the crosslinking process, which restricts molecular movements.

**Figure 6:**
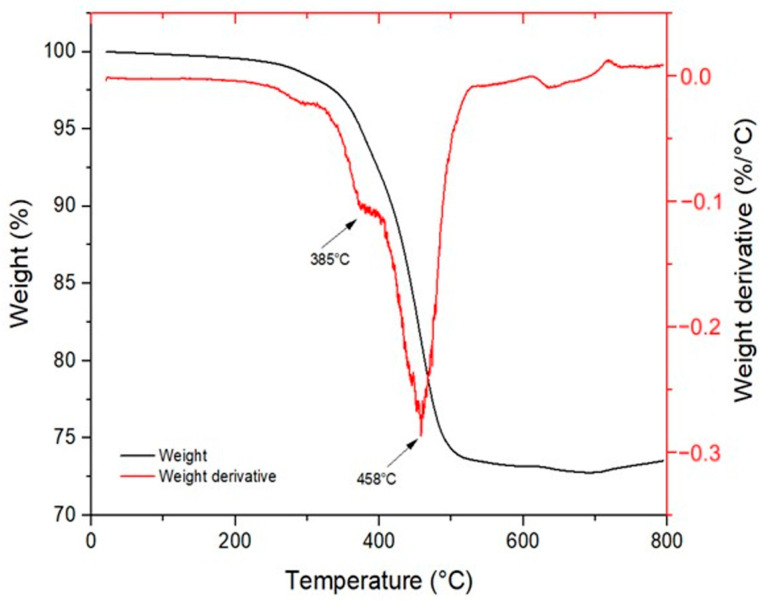
Thermogravimetric (TGA) analysis of the 6:3:1-75-20D resin.

In detail, several characteristic peaks were identified: the peaks at 2925 cm^−1^ and 2850 cm^−1^ correspond to the stretching vibrations of the CH_3_ and CH_2_ groups, respectively. The strong absorption at 1722 cm^−1^ is associated with the C=O stretching of ester groups. Peaks at 1636 cm^−1^ and 1620 cm^−1^ are related to the C=C stretching vibrations of alkenes. The bands at 1463 cm^−1^ and 1408 cm^−1^ are attributed to the bending vibrations of methyl groups. Peaks at 1269 cm^−1^, 1188 cm^−1^, 1050 cm^−1^, and 924 cm^−1^ are assigned to the symmetric and asymmetric stretching of C–O–C ester bonds. The absorption at 806 cm^−1^ corresponds to the out-of-plane bending vibration of the vinyl groups present in the acrylate moieties. The C=C stretching peaks show a significant decrease in intensity after curing, indicating the completion of the crosslinking reaction. Similarly, the 806 cm^−1^ peak, associated with the out-of-plane vibration of the vinyl group, decreases due to the consumption of double bonds in favor of newly formed C–C single bonds. The overall reduction in molecular mobility, a result of the formation of a three-dimensional polymer network, is also evident from the decreased intensity of the 2925 cm^−1^ and 2850 cm^−1^ bands. The thermal degradation curve was obtained through TGA analysis on the specimen containing 20 wt% of DCP. As shown in Figure 6, at 800 °C, the specimen retains approximately 75 wt% of its original mass, indicating that the polymeric matrix has completely degraded while the metallic filler remains stable, confirming the expected nominal filler content in the resin. The degradation process occurs in two main steps. The first degradation event appears at around 385 °C, corresponding to the fragmentation and decomposition of non-reacted triglycerides. The second step, occurring at 458 °C, is attributed to the decomposition of the remaining main polymer chains. The material demonstrates good thermal stability, with a 5% mass loss occurring at 288 °C. This behavior is indicative of a high crosslinking density achieved during the curing process. Moreover, the absence of a peak around 100 °C suggests that the specimen was completely dry, as no mass loss associated with water evaporation was detected.

**Figure 7:**
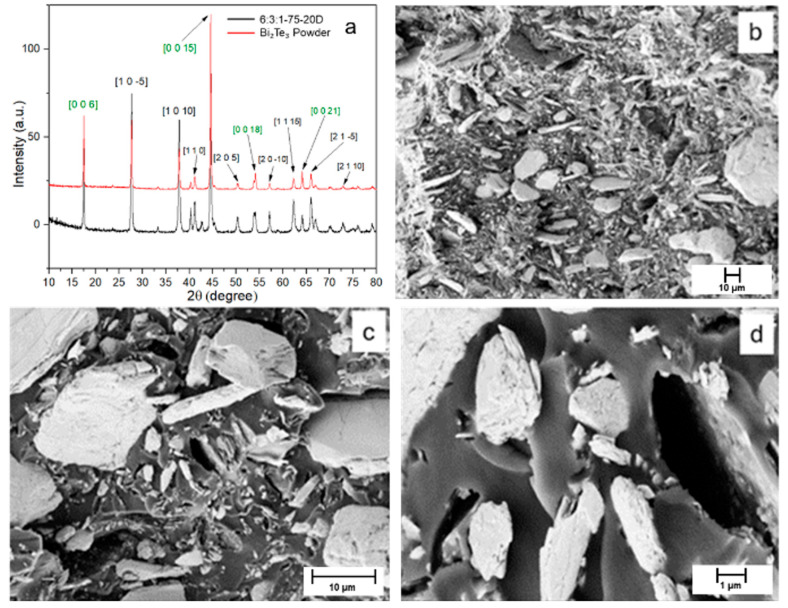
(**a**) XRD comparison between Bi_2_Te_3_ powder (red) and 3:2:1-75-20D resin (black) with highlighted characteristic peaks relative to the filler; (**b**–**d**) SEM images of the composite at different magnifications.

When comparing the XRD pattern of the powder in Figure 7a with that of the composite material, the peak positions are found to coincide. However, differences are observed in the peak intensities due to differences in the processing methods. In particular, four peaks (highlighted in green) corresponding to the [003] direction exhibit higher intensities, indicating a preferential crystallographic orientation of the Bi_2_Te_3_ filler within the matrix [44]. SEM analysis was performed on the cured 6:3:1-75-20D specimen, as shown in Figure 7b–d. The micrographs reveal a highly diverse bismuth telluride particle size, ranging from a few microns down to below 1 µm.

**Figure 8:**
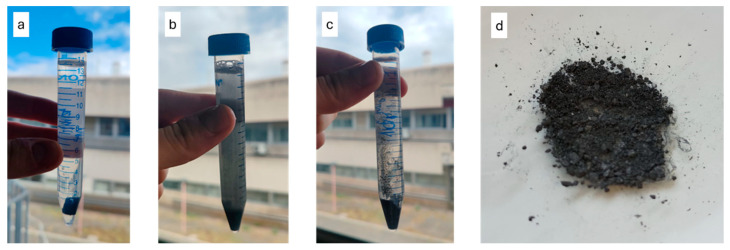
(**a**–**c**) Accelerated degradation test after 1, 4, and 7 days, respectively, showing complete degradation. (**d**) Bi_2_Te_3_ powder recovered from the degradation test.

A good distribution of the particles throughout the matrix is also noticeable, likely due to the ultrasonic dispersion treatment in acetone prior to mixing that determined the delamination of the bigger particles seen in Figure 2. The images further indicate that the overall porosity of the material is low, suggesting an effective curing process. They show how the filler particles (appearing as white regions) are well anchored within the polymeric matrix (black regions), confirming good interfacial adhesion between the matrix and the filler. To confirm the structural integrity of the Bi_2_Te_3_ filler after incorporation into the resin matrix, an additional X-ray diffraction (XRD) analysis was performed on the composite. The resulting diffraction pattern retained the characteristic peaks previously observed in the pure powder, including the prominent reflections along the [003] crystallographic direction and its higher-order multiples, indicating that the filler preserved its crystallographic orientation and was not altered during processing. The accelerated degradation test (Figure 8) showed that complete hydrolysis of the polymer occurred in approximately 7 days. Water can react with the long polymer chains, breaking them down into shorter fragments; this reaction is further accelerated by the presence of NaOH dissolved in the solution.

Degradation is favored in the presence of functional groups based on oxygen (O), nitrogen (N), or sulfur (S) along the polymer chains, as these groups induce a positive charge, higher than 0.3 electron charges, on the adjacent carbon atoms, thereby attracting water molecules and speeding up the hydrolysis process [45]. Among these kinds of groups, ester ones found in the AESO and PEGDA components are especially reactive, acting as the starting point for the degradation. The literature shows how hydrolysis follows second-order reaction kinetics, depending on the concentration of water and hydrolytic polymer bonds highly present in the AESO and PEGDA components [46]. Since partial degradation was achieved, meaning that the metallic filler remained undissolved and can be recovered through draining and drying, this indicates a good degradability of the material. Thus, the material could degrade naturally upon exposure to water from sources such as rain or humidity. Additionally, if the water contains salts, the degradation reaction could be further accelerated, as salts can act as catalysts, similarly to the role of NaOH in the test solution.

### 3.5. Thermoelectric Performance

**Figure 9:**
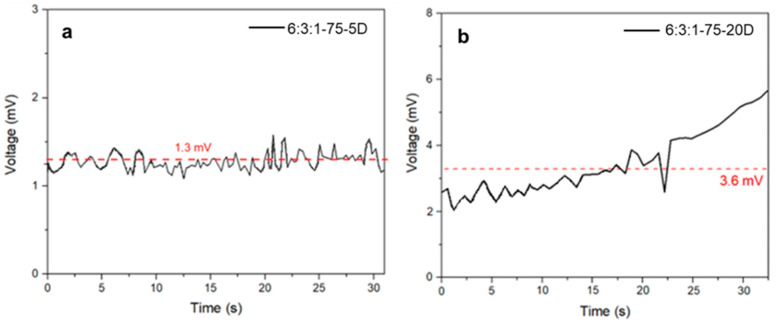
Thermoelectric analysis of 6:3:1-75-5D (**a**) and 6:3:1-75-20D (**b**) specimens. The red dotted line shows the average potential generated by the samples.

The thermoelectric tests were performed on the two DCP-based resin batches. Each specimen was subjected to a temperature difference of 70 °C. In Figure 9, the multimeter measurements are presented; by averaging the data collected over time, we obtained 3.6 mV and 1.3 mV, respectively, for the 20 wt% and 5 wt% DCP-containing resins. From the figures, it is noticeable that the highest DCP content resulted in the highest thermoelectric response. This trend suggests that increased crosslinking improves the thermoelectric behavior of the composite, likely by enhancing both filler connectivity and heat-transfer efficiency.

To better understand the signal characteristics and the nature of the voltage generation under thermal stress, a more detailed examination of the time-resolved voltage profiles was carried out. Contact resistance fluctuations may also contribute to the instability observed in the recorded voltage signals. Given that thermoelectric voltages typically lie in the microvolt to millivolt range, the measurements are highly sensitive to even minor variations in the quality of the electrical contacts between the electrodes and the sample surface.

In composite materials with a heterogeneous filler distribution such as Bi_2_Te_3_ particles embedded in a polymer matrix, local variation in conductivity and particle alignment can lead to uneven current pathways, resulting in signal noise and fluctuating potential readings [47]. The sample with lower crosslinking density (6:3:1-75-5D) exhibited a nearly steady voltage profile characterized by high-frequency fluctuations. This suggests that the thermal gradient across the sample remained relatively stable, but the internal conductive network was insufficiently developed. The lack of percolation between the thermoelectric domains likely limited charge carrier transport, resulting in low and noisy voltage output. Conversely, the more densely crosslinked sample (6:3:1-75-20D) showed a progressively increasing voltage trend with reduced relative noise, indicating a more coherent and stable thermoelectric response. This behavior may be attributed to improved filler–matrix interfacial contact, enhanced thermal transfer, and a better-formed percolative network facilitating charge transport through the bulk material [48]. The gradual voltage increase over time may also reflect delayed heat diffusion through the sample, especially in thicker or more compact specimens, where thermal equilibrium across the composite cross-section is achieved more slowly. Moreover, mechanical stresses induced during clamping, or differential thermal expansion between the matrix and filler, may lead to subtle microstructural rearrangements during heating, further contributing to temporal variations in the measured signal. Such time-dependent voltage evolution may also be indicative of gradual heat diffusion through the composite thickness, especially in more compact or denser samples, which delays the stabilization of ΔT and hence the thermoelectric output. Additionally, mechanical stresses generated during clamping or due to differential expansion between the filler and matrix can cause microstructural rearrangements during heating, further contributing to temporal signal variation. Using the averaged potential difference values, the Seebeck coefficient was calculated, with the results reported in Table 4 along with the sheet resistance, resistivity, thermal conductivity, and the figure of merit ZT, also compared with the literature.

While the overall densities of the 5 wt% and 20 wt% DCP specimens are similar, the markedly improved thermoelectric response of the latter is likely attributable to a higher crosslinking density. The increased amount of DCP probably leads to a more robust and coherent polymer network, which enhances the structural integration and interfacial contact between Bi_2_Te_3_ particles and the surrounding matrix. This improved interface facilitates more continuous and stable charge transport pathways, resulting in better electrical percolation and a higher Seebeck coefficient [51].

When comparing our results with the literature, pure Bi_2_Te_3_ typically exhibits Seebeck coefficients ranging from 100 to 200 μV/K, depending on carrier concentration and microstructural quality [14]. Our composites, which reach a maximum of 51 μV/K, perform significantly lower, as expected, due to the insulating nature of the bio-based matrix and the moderate filler content. However, when compared with other Bi_2_Te_3_ polymer composites, such as those reported in the literature which show Seebeck values in the range of 20–80 μV/K depending on composition and matrix type, our material exhibits comparable performance [52]. Furthermore, in the work of Su et al. [49], a Seebeck coefficient approximately three times higher was achieved using Bi_0.5_Sb_1.5_Te_3_ with 90 wt% filler and thermal annealing post-treatment at 450 °C for 6 h determining the decomposition of their polymeric matrix around the filler. A thermal post-treatment (performed at a compatible temperature with the bio-resin) could be considered for our composites to promote a partial rearrangement of the filler within the matrix. This may help reduce the mean distance between the Bi_2_Te_3_ particles, enhancing interparticle connectivity and consequently lowering electrical resistivity, with the potential to improve the Seebeck coefficient.

The figure of merit (ZT), calculated using Equation (2), remains within the expected range for thermoelectric composites based on insulating polymer matrices [50]. Although the low thermal conductivity of the matrix is favorable, the overall ZT remains limited due to high electrical resistivity, which is dominated by the polymeric matrix.

Several factors may contribute to the reduced Seebeck and ZT values. As shown in Figure 7, the Bi_2_Te_3_ particles display a heterogeneous size distribution and loose packing, which likely hinders electronic percolation. These inhomogeneities lead to uneven charge transport routes and insufficient conductive network formation. Furthermore, the insulating matrix introduces interparticle gaps, further limiting electron flow. While such discontinuities can also reduce thermal conductivity, the reduction is not sufficient to compensate for the loss in electrical performance. Consequently, the resulting ZT remains modest.

## 4. Conclusions

In this work, the development of thermoelectric composite resins based on an AESO–PEGDA–PETA polymeric matrix filled with bismuth telluride was successfully achieved. The photocurable and thermal curing strategies, using BAPO and DCP initiators, respectively, allowed the fabrication of specimens with tunable curing kinetics and mechanical stability. Although the high filler content hindered the direct 3D printing process via DLP without further optimization, thermal curing enabled the production of dense and well-dispersed composites. Material characterization, including density measurements, FTIR, SEM, and TGA analyses, confirmed the formation of a stable, highly crosslinked network with controlled filler dispersion and good thermal stability. In particular, the TGA analysis revealed a two-step degradation behavior at approximately 385 °C and 450 °C, indicating the composite’s suitability for use under moderate thermal stress. Accelerated degradation tests demonstrated the degradability of the matrix, suggesting potential environmental compatibility for future applications and the possible complete recovery of the Bi_2_Te_3_ filler. The thermoelectric tests showed that a higher DCP content, leading to a lower porosity, significantly improves the thermoelectric response of the composite; in fact, the formulation containing 20 wt% DCP achieved a Seebeck coefficient of 51 μV/K, a value comparable to those reported in similar Bi_2_Te_3_–polymer composites. Despite operating under mild conditions and without complex thermal post-treatments, the achieved Seebeck coefficients are indeed low with respect to traditional TEGs, but promising compared with the literature benchmarks. Overall, the obtained results open the way for further optimization of both the resin formulation and processing parameters, aiming to enhance the thermoelectric performance while maintaining the advantages of simple, scalable manufacturing. Given their processability and partial degradability, these materials show potential for integration as thermoelectric coatings, thin films, or structural layers for waste heat recovery in low-temperature industrial systems, smart packaging, or autonomous low-power sensors. Their applicability in areas where moderate thermal gradients are present, such as mechanical housing, pipelines, or electronics enclosures, makes them attractive for sustainable, embedded energy-harvesting solutions.

## Figures and Tables

**Table 1:** Overview of the produced formulations and relative nomenclature, with matrix composition, filler loading, and curing agent type and concentration.

Formulation	PEGDA–AESO–PETA Weight Ratio	Filler (wt%)	BAPO (wt%)	DCP (wt%)
5:5:1-25-1B	5:5:1	25	1	-
5:5:1-50-1B	5:5:1	50	1	-
5:5:1-75-1B	5:5:1	75	1	-
5:5:1-75-2B	5:5:1	75	2	-
6:3:1	6:3:1	0	1	-
6:3:1-75-1B	6:3:1	75	1	-
6:3:1-75-2B	6:3:1	75	2	-
6:3:1-75-5D	6:3:1	75	-	5
6:3:1-75-20D	6:3:1	75	-	20

**Table 2:** DLP printing parameters used for BAPO-based resins.

Sample	Bottom Layers Exposure Time (s)	Normal Exposure Time (s)	Layer Height (µm)
1	120	90	50
2	180	120	50
3	360	200	50
4	360	200	100

**Table 3:** Experimental and theoretical densities of the relative specimens with different DCP contents and different curing temperatures.

Specimen	Experimental Density	Theoretical Density
6:3:1-75-5D cured at 100 °C	3.07 g/cm^3^	3.08 g/cm^3^
6:3:1-75-20D cured at 100 °C	3.6 g/cm^3^	3.08 g/cm^3^
6:3:1-75-20D cured at 120 °C	1.85 g/cm^3^	3.08 g/cm^3^

**Table 4:** Seebeck coefficient, sheet resistance, resistivity, thermal conductivity, and ZT calculated from the thermoelectric analysis with a temperature gradient of 70 °C (n.a. = not available).

DCP (%w)	Seebeck Coefficient (µV/K)	R_s_ [kΩ/□]	ρ [kΩ·m]	K [W/K·m]	ZT
5	19 ± 2	3567	10.701	0.43	2.42 × 10^−11^
20	51 ± 4	2138	6.414	0.47	2.66 × 10^−10^
100% Bi_2_Te_3_ [49]	141	n.a.	1.1 × 10^−4^	(0.53–0.55)	(0.7–0.11)
T. Rodrigues-Marinho et al. [50]	(15–36)	n.a.	n.a.	n.a.	(10^−6^–10^−15^)

## Data Availability

The original contributions presented in this study are included in the article. Further inquiries can be directed to the corresponding author.
